# Congenital toxoplasmosis: an in-depth density-equalizing mapping analysis to explore its global research architecture

**DOI:** 10.1186/s13071-015-1263-x

**Published:** 2015-12-21

**Authors:** Dörthe Brüggmann, Vanessa Handl, Doris Klingelhöfer, Jenny Jaque, David A Groneberg

**Affiliations:** Department of Obstetrics and Gynecology, Keck School of Medicine of USC, Los Angeles, CA USA; Department of Female Health and Preventive Medicine, Institute of Occupational Medicine, Social Medicine and Environmental Medicine, Goethe-University, Frankfurt, Germany

## Abstract

**Background:**

Toxoplasmosis endangers the unborn child if its infectious agent - toxoplasma gondii - is transmitted transplacentally during pregnancy. Although this condition occurs in all parts of the world and represents a major public health burden, no detailed knowledge on the global research architecture of congenital toxoplasmosis is available thus far. Hence, it was the aim of this study to assess the related global research activity over the past 110 years.

**Methods:**

We employed the NewQIS platform, which combines established scientometric and socioeconomic analysis tools with novel visualizing techniques such as density equalizing mapping projections.

**Results:**

In the Web of Science, 13,044 congenital toxoplasmosis-related items published between 1900 and 2012 were identified. These were issued by 26,483 authors originating from 125 countries. The US was the dominating nation (38.7 % of total scientific output), followed by France (10.9 %) and Great Britain (9.2 %). The US also led the ranking in regards to semi-qualitative parameters (total citations, country-specific h-indices and citation rates). When research activity was related to economic figures, the ratio of total toxoplasmosis publications to the total GDP listed Switzerland first with an average of 589.35 toxoplasmosis-related publications per GDP in 1000 Bio US-$, followed by France (545.16), the UK (486.13) and Brazil (431.84) and the US (311.11). The relation of toxoplasmosis-specific publications to the economic power indicator GDP per capita in 1000 US-$ revealed that the US was ranked first with 97.65 toxoplasmosis-related publications/GDP per capita in 1000 US-$, followed by Brazil (85.95). Subject area analysis indicated a relative shortage of studies that addressed pharmacological or public health aspects of congenital toxoplasmosis.

**Conclusions:**

This study is the first in-depth approach to sketch a global picture of the congenital toxoplasmosis research architecture. In contrast to other fields of biomedical research, not only high-income countries play a major role regarding congenital toxoplasmosis research but also countries such as Brazil that have a high incidence of congenital toxoplasmosis.

## Background

Toxoplasmosis, caused by the obligate intracellular protozoan Toxoplasma gondii (T. gondii), is a widespread zoonosis of great importance worldwide [1]. The condition is usually contracted by consumption of raw or undercooked meat containing tissue cysts, or by ingesting food or water contaminated with oocysts [1]. Infection during pregnancy poses a major risk to the fetus due to transplacental transmission [2]. Populations at risk for congenital toxoplasmosis (CT) include immunocompetent women when become newly infected in pregnancy or challenged with atypical parasite strains as well as immunosuppressed mothers with HIV/AIDS [3]. The risk of vertical transmission increases during the course of the pregnancy whereas the severity of fetal sequelae declines and depends on the virulence of the T. gondii genotype [2]. Classic fetal manifestations include chorioretinitis, hydrocephalus and intracranial calcifications. In up to 80 % of cases, the infection remains asymptomatic after birth, but infants will develop learning and visual disabilities later in life [2, 4].

Many factors impact the epidemiology of T. gondii infections such as environmental conditions (e.g. climate), local density of felines, management of livestock and habits of meat consumption. Average incidence rates of 4 T. gondii infections per 1000 pregnancies are reported for European countries [5–8], while France is having the highest incidence of congenital toxoplasmosis based on 2700 documented seroconversions in pregnant women annually [9]. Other regions with a significant T. gondii infection incidence include South America (e.g. incidence rates of seven infections per 1000 pregnancies in Argentina and up to 15 infections per 1000 pregnancies in Colombia) and sub-saharan Africa [7, 10]. Numerous genotypes of T. gondii have been identified so far. These differ regarding their global distribution, prevalence and virulence. Overall, three major subtypes (I, II, III) account for 95 % of isolates in North America and Europe. 5 % of subtypes are atypical genotypes of great genetic diversity [11, 12] such as the BR 1 to 4 haplogroups detected in Brazil [13]. Atypical toxoplasma create a more virulent parasite population in South America so congenital toxoplasmosis presents more severely compared to Europe. A large meta-analysis and a prospective cohort study showed a higher risk of ocular lesions for Brazilian and Columbian than European children (47 % versus 14 %). Further, these lesions were larger, more numerous and more likely to affect the retina [14–16].

Research on congenital toxoplasmosis is a relatively new field. T. gondii was initially described in 1908 [17] and identified as the causative agent for neonatal encephalomyelitis in the 1930s [18]. The mode of infection by vertical transmission was published a decade later. Important discoveries regarding the diagnosis of congenital toxoplasmosis, the life cycle and the virulence mechanisms of T. gondii were made in the 1960s and 1970s based on the availability of novel immunological, cell culture based, and molecular biological research methods [19, 20]. In the 1990s and the new millennium, sophisticated genetic approaches allowed the characterisation of the numerous T. gondii genotypes worldwide [20, 21].

The omnipresent and highly prevalent parasite represents a threat to every non-infected pregnant women around the globe [22]. The economic burden of toxoplasmosis is high. The costs - primarily attributed to congenital toxoplasmosis - are estimated at nearly $8 billion yearly in the US alone [23]. Nevertheless, no standardised prevention measures such as vaccinations or an optimal curative therapy have been developed so far [24]. Also, public health interventions such pre- and neonatal screening programs or surveillance networks have been implemented in just a few nations globally [2]. Considering these unsolved challenges, further research is necessary. Detailed knowledge on the global research architecture of congenital toxoplasmosis is crucial for planning scientific efforts, individual scholarship and performance assessment in the field, but becomes challenging due to the vast number of related scientific publications available. Thus, scientometric methods can be utilized for the systematic and reliable analysis of journal articles. For this study, we employed the New Quality and Quantity Indices in Science (NewQIS) platform [25], which combines scientometric methods and advanced visualizing procedures such as density equalizing mapping, to assess the global research on congenital toxoplasmosis over the past 110 years. Therefore, we focused on quantitative and qualitative aspects of the research output, geographical and chronological developments, existing research networks and socio-economic benchmarking.

## Methods

### NewQIS protocol

The New Quality and Quantity Indices in Science (NewQIS) platform encompasses a combination of established economic and scientometric analysis tools together with novel visualizing techniques such as density equalizing mapping projections (DEMP). It was established in 2009 and described in detail previously [26, 27].

### Data source

We used the Web of Science database (WoS core collection, Thomson Scientific) for data collection [28, 29]. This index database covers all CT-related scientific literature authored since 1900. It provided quantitative data regarding the overall research output and enabled us to assess citation parameters supplied by the Citation Report.

### Search strategy

Specific combinations of search terms containing congenital*, fetal*, prenatal* toxoplasm* and gondi* were used. The asterisk was used as a wildcard for considering variant forms of spelling [30]. We performed a “title” not a “topic” search. This process minimized the inclusion of off-topic and therefore unspecific publications in our analysis. We restricted our investigation to the period from 01.01.1900 to 31.12.2012 to avoid incomplete data acquisition since the year 2013 was not completed at the time the study was performed.

### Data analysis and categorization

After retrieval of metadata in Plain Text Format, bibliographic details were sorted in an interim database and analysed according to specific and semi-qualitative criteria. Quantitative criteria included countries of origin, languages, document types, citations, cited references, subject categories and year published. From extracted metadata, CT-specific modified H-indices and citation rates, were calculated as semi-qualitative variables. The H-index (HI) was developed by Jorge Hirsch in 2005 and is a recognized parameter for the scientific quality of publications [31]. To measure the chronologic evolution of the total global CT-associated research output, we performed a regression analysis and calculated the coefficient of determination (r^2^). r^2^ quantified the slope of publication increase in correlation to two defined time periods: (1) 1900 – 2012 (the total time period of our investigation) and (2)1990 – 2012 (time period with a visible pronounced increase in publication activity).

### Density equalizing mapping

After raw data transfer to excel charts, the results were illustrated in diagrams and visualized by DEMP as previously described in other NewQIS studies [32–35]. Based on the algorithm of Gastner and Newman [36], the territories of the countries and their borders were resized in proportion to the analysed variable, e.g. number of publications or citations, and a global sketch depicting specific aspects of the CT-related research activity was created.

### Economic analysis

In order to analyze a country’s scientific productivity in the context of its economic resources and manpower, we related its research output to recognized economic benchmarks such as the gross domestic product (GDP) per capita, and to the total economic power index GDP per 1000 billion US-$. Economic statistics were obtained from the World Economic Outlook Database of the International Monetary Fund and the statistical yearbook of the Federal Office of Statistics, Germany [37, 38]. The data for the comparison of GDP per inhabitant were taken from the World Development Indicators of The World Bank [39, 40]. Further, we used the classification issued by the World Bank to categorize the investigated countries into high-income, upper-middle-income, lower-middle-income and low-income countries (http://data.worldbank.org/country/).

### Analysis of collaborations

To investigate toxoplasmosis-related research collaborations in a global context, all affiliations of the toxoplasmosis-related publications were analysed as earlier described for other diseases [41, 42]: In brief, if at least two authors originating from different countries contributed to one CT-related publication, this relationship was defined as a collaboration. To visualize the productivity of collaborations for single pairs of countries, a vector was generated. Its width and shade of grey reflect the number of documented collaborative publications [41, 42].

### Gender analysis

We investigated the distribution of male and female authors and identified the countries where these researchers were conducting their work. Only countries with more than 300 authors, whose gender could be identified in over 60 % of cases were included. In order to identify the authors’ gender, we utilized online name databases (http://www.vornamen-weltweit.de/ and http://www.namepedia.org) and performed a manual search employing websites, corresponding addresses and social networks.

## Results

### General parameters

We identified a total of 13,044 CT-related publications issued between 1900 and 2012. 10,026 of these items were articles. The publications were authored by 26,483 individuals originating from 125 countries and published in 1600 journals. 94 % of the examined works were written in English (12,257 publications), 2.1 % in French (278 publications) and 1.6 % in German (211 publications). 93 of identified CT-related publications were written in Spanish, 79 in Portuguese, 32 in Russian, 23 in Italian, 17 in Turkish, 15 in Polish and eight in Czech.

The first article indexed by WoS was published in 1913. Overall, we could document a very modest annual publication output (maximum of 20 items per year) from 1940 to 1960. This publication activity increased steadily after 1960 and was followed by a steep incline after 1990. In detail, we identified 91 publications for 1989. In 1993, this volume increased to 317 and the trend continued: In 2005, 536 items were issued, 806 in 2009 and 877 in 2012. Of the 13,044 articles that were published in total, the majority of 11,226 (equal to 86.1 %) were published between 1990 and 2012.

**Fig. 1:**
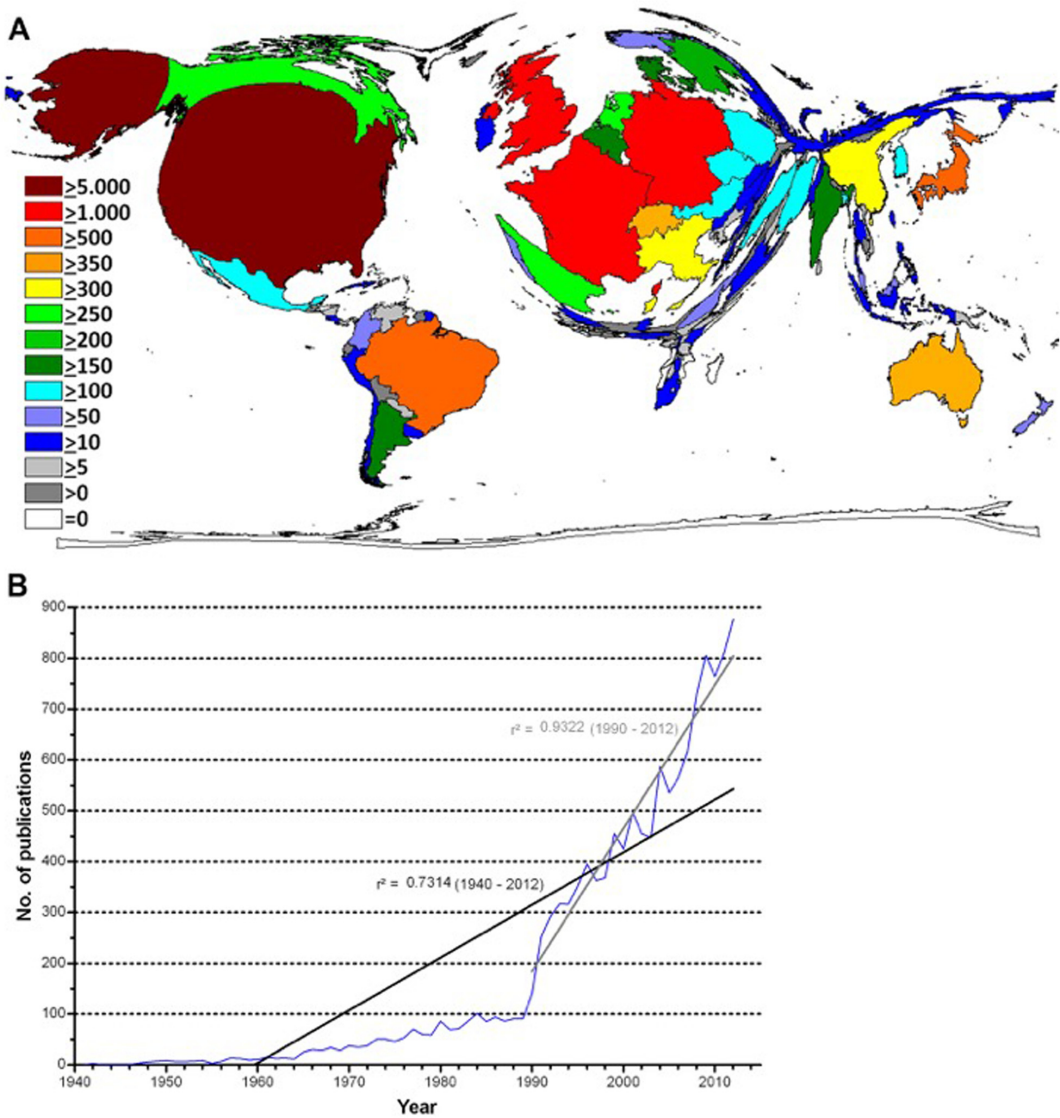
Number of publications. **a** Density equalizing map of the global toxoplasmosis research activity assessed by publication output between 1900 and 2012. Colours and territorial sizes indicate numbers of toxoplasmosis publications per country. **b** Number of published items per year

We quantified the slope of the increasing publication output by regression analysis. For the period between 1940 and 2012, we found a r^2^ of 0.7314 compared to a r^2^ of 0.9322 for the period between 1990 und 2012, which documented a noticeable incline in research output (Fig. 1b). Also, the number of participating authors per publication increased over the time. While there was an average of less than three authors involved per article between 1966 and 1982, this number grew to more than six in 2012 (data not shown).

### Country-specific analysis

With 5045 publications, the United States (US) published most items (38.7 % of the total volume) followed by France (1425 articles, 10.9 %), the United Kingdom (UK) (1204 articles, 9.2 %), Germany (1051 articles, 8 %) and Brazil (973 articles, 7.5 %). Japan (553 articles), Australia (397 articles), Switzerland (372 articles), Italy (328 articles) and China (319 CT-specific publications) were among the ten top ranked countries. In a global context, the US played the dominanting role regarding CT-specific research output. Also, Western Europe, Brazil and Japan occupied major territories on the world map. The majority of Asian nations including Russian, African and South American countries (with the exception of Brazil) were represented by only minimal areas (Fig. 1a).

**Fig. 2:**
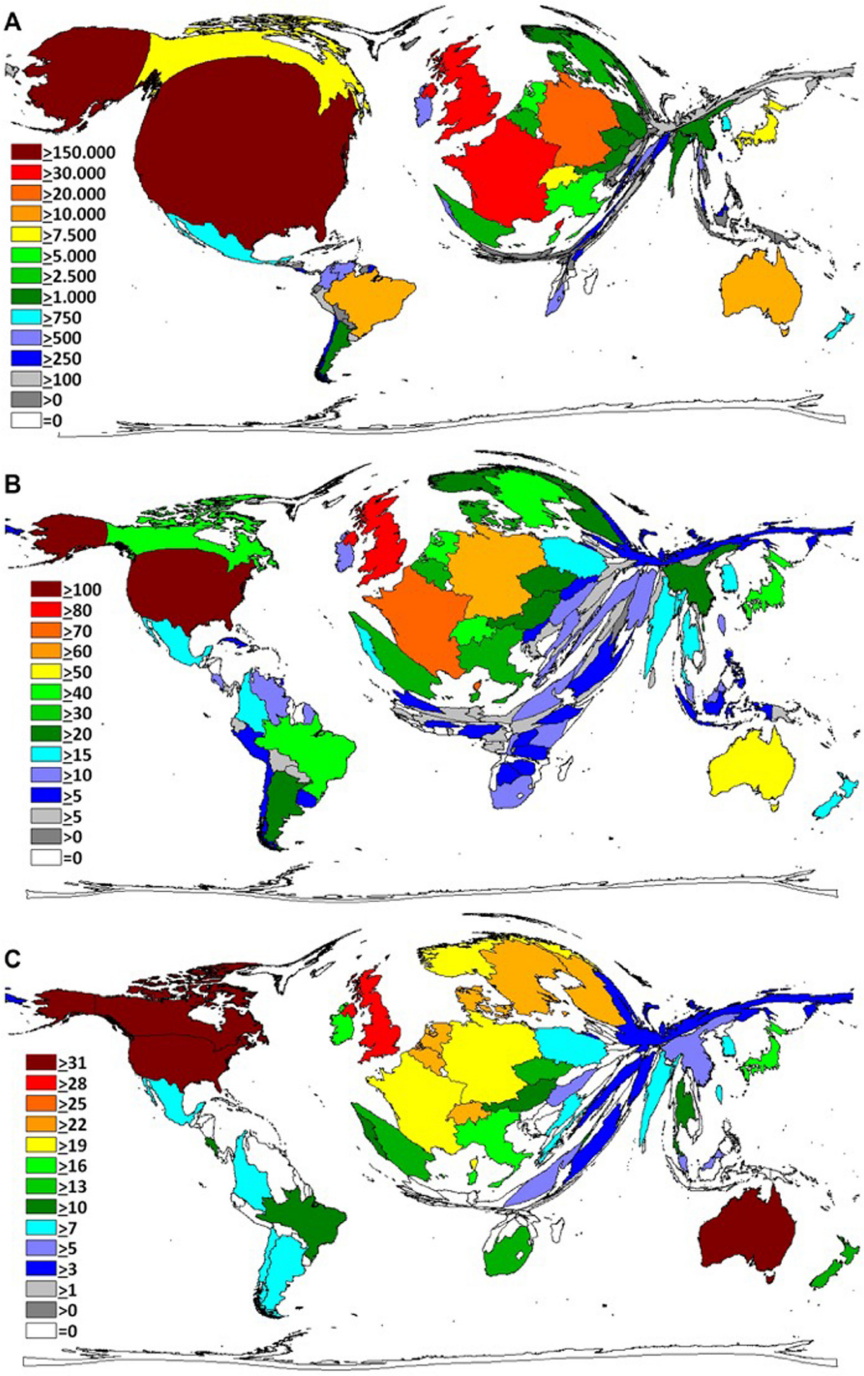
Density equalizing maps of the global toxoplasmosis research quality. **a** Number of citations per country. **b** Toxoplasmosis-specific h-indices. **c** Citation rate. Colours and territorial sizes indicate levels of toxoplasmosis-specific parameters

Regarding total citation number analysis, the US hold position one with a total of 159,819 citations. It was followed by the UK (35,383 citations), France (30,775 citations), Germany (22,015 citations), Australia (12,853 citations) and Brazil (11,674 citations) (Fig. 2a). Analysing the modified H-index for countries, the highest HI was attributed to the US (HI = 149) followed by the UK (HI = 83), France (HI = 77) and Germany (HI = 62). Brazil was ranked 7 with an HI of 47, followed by Australia (HI = 53) and Switzerland (HI = 49) (Fig. 2b). In category “citation rate” (CR) a change in the ranking was present with Australia reaching the highest level of citations per CT-specific publication (32.4). It was followed by the US (CR 31.7), Canada (CR 31.6), the UK (CR 29.4), France (CR 21.6) and Germany (CR 20.9) (Fig. 2c).

### Economic analysis

When focussing at the gross domestic product as an indicator for total economic strength and its relation to the CT research output of single countries, all of the ten most productive countries – except Australia and Switzerland- belonged to the states with the highest total GDP among the world (Table 1). The majority of these nations represented high-income countries according to the World Bank whereas the upper-middle-income countries Brazil and China were the two exceptions.

**Table 1:** Socio-economic analysis of toxoplasmosis research of the ten most active countries. Sources for GDP and GDP per capita in 2012: [37–40]

Country	Number of publications	GDP in 1000 Bio US-$	Publications/GDP in 1000 Bio US-$	GDP per capita in 1000 US-$	Publications/GDP per capita	Type of country
Switzerland	372	0.6312	589.35 (1)	78.93	4.71 (10)	HI
France	1425	2.6139	545.16 (2)	39.76	35.84 (4)	HI
UK	1204	2.4767	486.13 (3)	38.65	31.15 (5)	HI
Brazil	973	2.2531	431.84 (4)	11.32	85.95 (2)	UMI
US	5054	16.2446	311.11 (5)	51.76	97.65 (1)	HI
Germany	1051	3.4295	306.45 (6)	42.60	24.67 (6)	HI
Australia	397	1.5417	257.50 (7)	67.44	5.88 (9)	HI
Italy	328	2.0141	162.85 (8)	33.81	9.70 (8)	HI
Japan	553	5.9603	92.78 (9)	46.55	11.88 (7)	HI
China	319	8.221	38.80 (10)	6.09	52.35 (3)	UMI

*HI* high-income, *UMI* upper-middle-income

The ratio of total publication number to total GDP was used to determine the relative focus of a country on toxoplasmosis research with regard to its overall economic capability. Switzerland was listed first with an average of 589.35 CT-related publications per GDP in 1000 Bio US-$. It was followed by France (545.16 publications/GDP in 1000 Bio US-$), the UK (486.13 publications/GDP in 1000 Bio US-$) and Brazil (431.84 publications /GDP in 1000 Bio US-$). The US was ranked fifth (311.11 CT-related publications per GDP in 1000 Bio US-$) and Germany sixth (306.45 publications per GDP in 1000 Bio US-$). The two Asian countries Japan and China were ranked last (92.78 and 38.80 publications per GDP in 1000 Bio US-$, respectively) indicating a relatively low interest and financial dedication in toxoplasmosis-focussed research despite high absolute numbers of publications i.e. in Japan. When the total number of CT-related publications was related to the relative economic power indicator GDP per capita in 1000 US-$, the US was again ranked first with 97.65 toxoplasmosis-related publications/GDP per capita in 1000 US-$, followed by Brazil (85.95 publications/GDP per capita in 1000 US-$). Here, Switzerland leading the absolute ranking due to its total GDP was found in the last position with 4.71 publications/GDP per capita in 1000 US-$ (Tab. 1).

### Subject area analysis

**Fig. 3:**
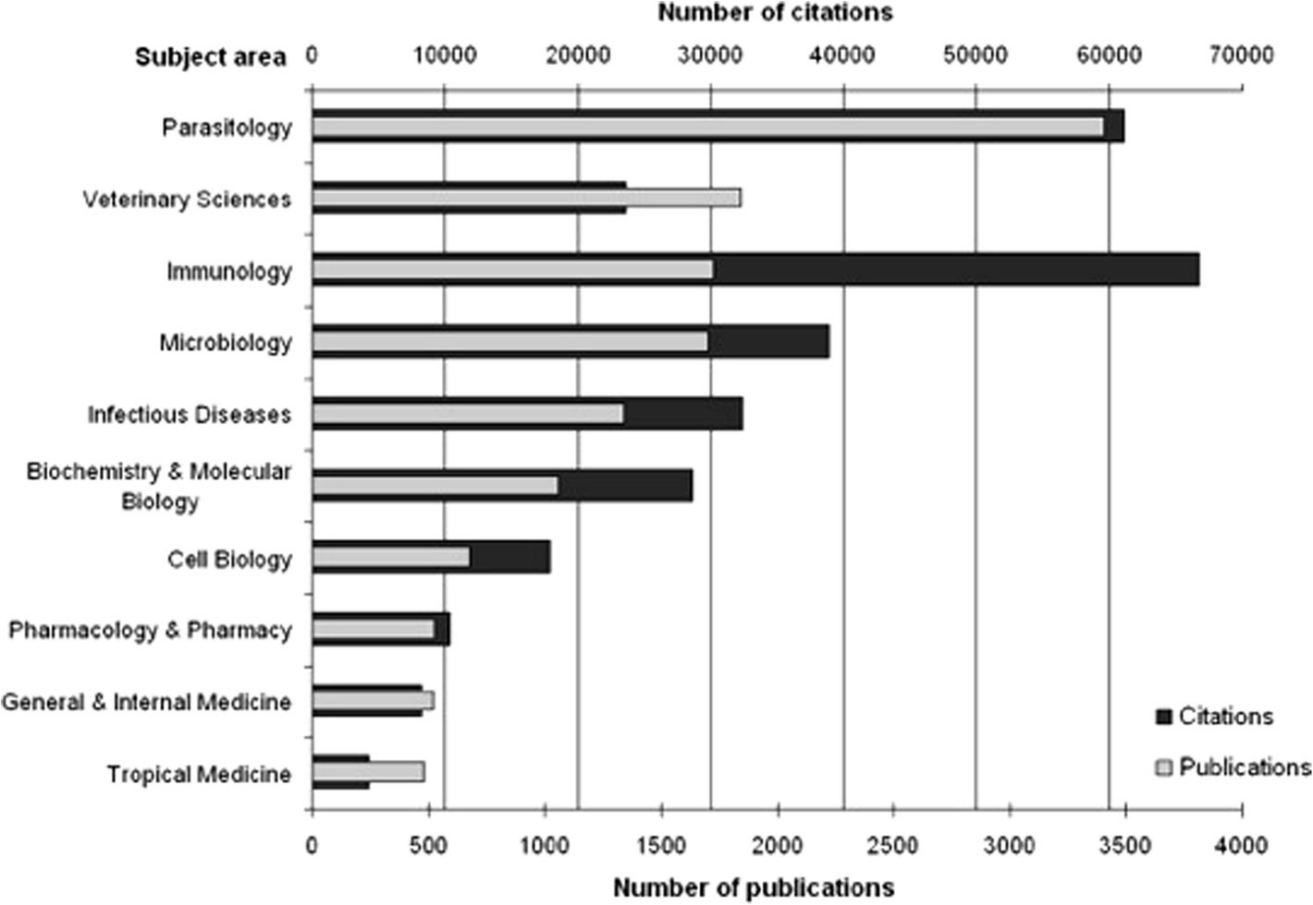
Leading subject areas in the field of toxoplasmosis research

The categories “Parasitology” and “Veterinary Science” represented the leading areas of CT research. 3406 publications were assigned to “Parasitology”, 1845 publications to “Veterinary Science”. “Immunology” (1724 publications) and “Microbiology” (1702 publications) followed at positions three and four. Publications in relatively new scientific fields such as “Molecular Biology” or areas of basic science were ranked lower at positions six and seven. With regard to total citation numbers, the leading subject areas were “Immunology” (66,767 citations), “Parasitology” (61,111 citations) and “Microbiology” (38,878 citations) (Fig. 3).

**Fig. 4:**
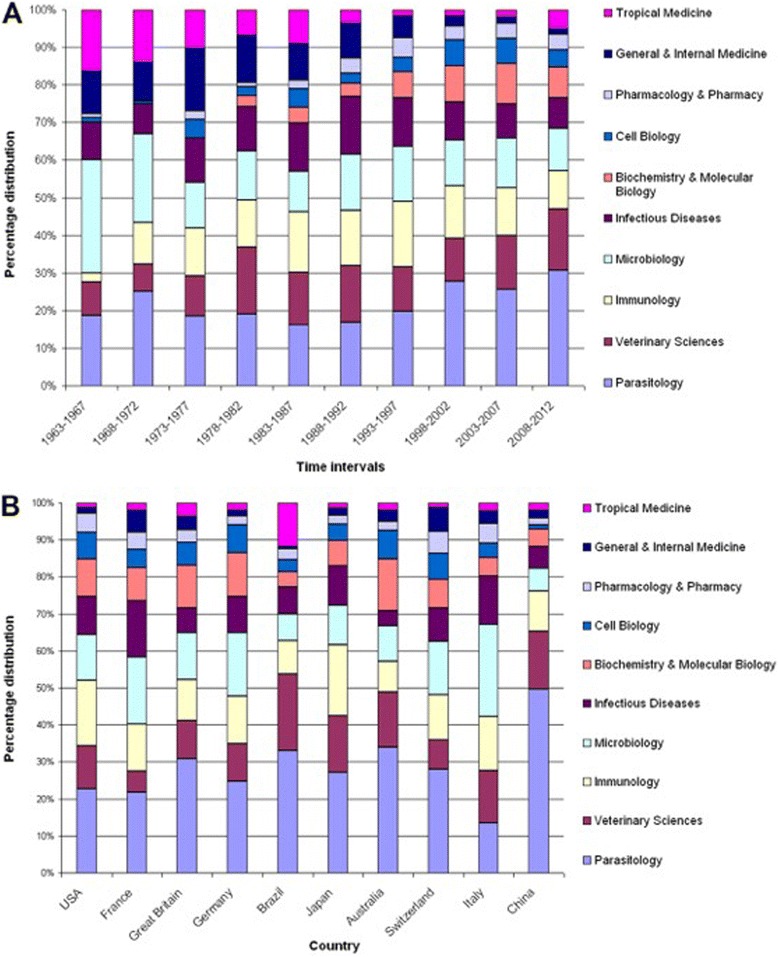
Subject area analyses. **a** Relative proportions of subject areas in 5-year intervals between 1963 and 2012. **b** Relative proportions of subject areas in most active countries

The percentage distribution of the ten most frequent subject areas was analysed in regard to 5 year-periods from 1963 onwards (Fig. 4a). The category “Tropical Medicine” increased in absolute numbers from 1963 to 2012 while its relative proportion decreased between 1963 and 2002. A similar trend was visible for the category “Microbiology”. Authors started to publish articles in the new categories “Cell Biology” after 1973 and in “Biochemistry and Molecular Biology” after 1978. The category “Biochemistry & Molecular Biology” increased both in absolute and relative numbers indicating an increasing focus of this area of CT research over the years.

When analysing the relative and absolute levels of assigned subject categories with regard to the most active countries in toxoplasmosis research, “Parasitology” took a lead position in all countries except Italy (13 %). In the US 24 %, in France 23 %, in the UK 30 %, Germany 26 %, in Brazil 36 %, in Japan 30 % and in China 53 % of all toxoplasmosis publications were primarily assigned to this subject area. In four countries, “Microbiology” was ranked second (France 19 %, UK 12.5 %, Germany 18 %, and Switzerland 15 %) whereas “Veterinary Science” was ranked second in Brazil, Australia and China indicating a difference in secondary research focuses between these two country groups. “Cell Biology” had a share between 1 and 8 % in each country. “Pharmacology & Pharmacy” never exceeded 6 %. The category of “Tropical Medicine” ranked in the positions seven to ten for all other countries (i.e. USA 1.5 %, France 2.1 %, Japan 1.6 %, Australia 2 %, Italy 2.1 % and Switzerland 1.3 %) with the exception of Brazil (Fig. 4b).

### International toxoplasmosis collaborations

**Fig. 5:**
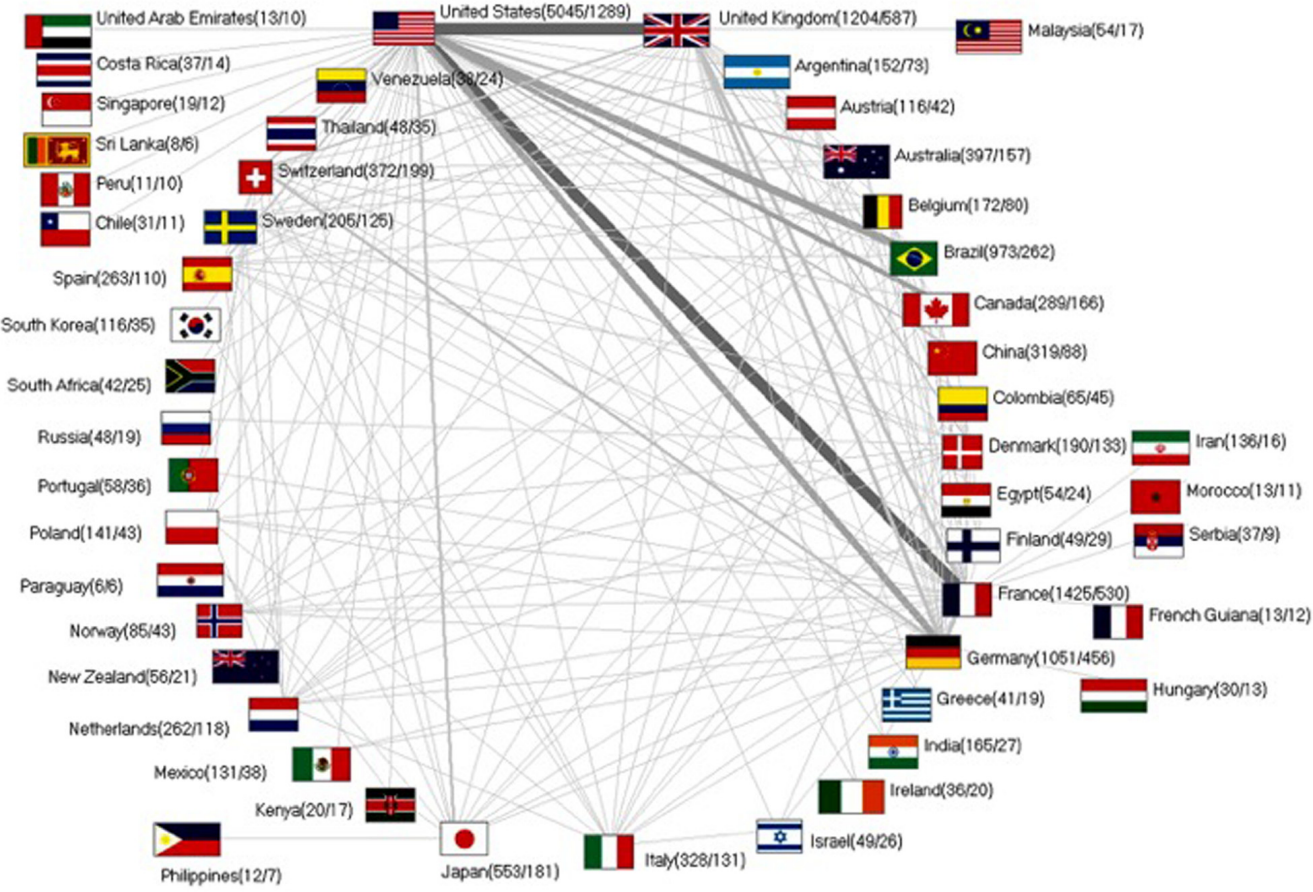
Toxoplasmosis research collaborations between countries. Greyscale and bar thickness indicate intensity of collaborations. Ciphers in brackets (total publication numbers / number of collaborative publications)

Scientists from the US and France issued the first collaborative publication in 1973. The number of joined publications increased consistently until 2012. The assessment of international collaborations demonstrated a leading position of the US among the global scientific community dedicated to CT-related research. Authors based in the US participated in 1289 collaborative CT publications, 255 of those were collaborations with the UK. The second highest number in bilateral collaborations was established between the US and France (234 co-operations), followed by the US and Brazil (171 co-operations), the US and Germany (146 co-operations) and the US and Canada (115 co-operations) (Fig. 5).

### Gender analysis

We identified 26,483 authors who participated in CT-related research; 48.8 % of those could be identified regarding their gender (n: 12,924). Generally, males were over-represented with 7884 authors (61 %) compared to 5049 female authors (39 %). In almost all countries,the number of male scientists was higher than their female counterparts but we documented two exceptions: Brazil and France. Here, female scientists clearly dominated. We found 164 female authors working in France compared to 141 male scientists; in Brazil, 343 female researchers compared to 279 males.

## Discussion

This study is the first in-depth analysis of the global congenital toxoplasmosis research activity regarding quantitative and semi-qualitative aspects and in relation to socio-economic variables. In total, 13,044 CT-related publications were issued from 1900 to 2012. Until 1960, the annual research output was low, followed by a steady growth until 1990 and a steep incline afterwards. This publication pattern is characteristic for most biomedical research, studies on MRSA [34] or influenza [43] provide examples. The modest research activity in the first 60 years can be explained with the newness of the field. The parasite had been known for only 50 years, its clinical impact on human health was not fully discovered yet and related research was not a priority for the scientific community. Also, the overall publication activity was lower; researchers were not mainly assessed by their publication merits. Beginning of the last century, many articles were published in other languages than English. These items were under-represented in our search since the WoS indexes mainly English publications. In the 1960s, the growing reasearch output correlated with milestones in CT research like the identification of the parasite’s life cycle, pathogenetic mechanisms and relevant clinical implications of CT (e.g. the discovery of the full scope of sequalae in humans). It is remarkable that 86.1 % of all CT-related publications were issued after 1990. This rapid incline might be related to the inclusion of additional publication types in the WoS since 1991 [44]. Thus, publications other than articles were picked up by our search. In the 1990s, the widespread use of novel research techniques broadened the opportunities to conduct and publish CT-related research. Additionally, research networks such as the “European Research Network on Toxoplasmosis” were founded and surveillance programs had been in place for almost 20 years (e.g. in France). We assume this wealth of epidemiological data and international collaborative efforts contributed significantly to this strong CT-related publication output.

The US played the leading role in the global arena of CT research: It published the highest number of items, obtained most citations, had the highest h-index and was home to most institutions conducting CT research. These findings align with the study by Groneberg-Kloft et al. [45], who analyzed the overall publication output of the biomedical field from 1961 to 2007. Here, the US issued 1,893,800 of 5,527,558 publications and was the most productive country in regards to research on 21 organ systems [45]. This dominance might be attributed to the fact that the US is allocating major resources to research in general. In 2012, the nation dedicated 2.9 % of its 16.244,6 billion US-Dollars GDP to scientific purposes [37–40]. While the UK and Germany are also known key players in the global arena of biomedical research [45] and contributed 9.2 and 8 % of CT-specific publication output respectively, our study provided the remarkable result that France and Brazil were in similarly leading positions. This finding can be explained by the fact that the disease is of great national interest in both countries: France has the highest incidence of CT in Europe, mainly due to the common consumption of undercooked meat [2]. Tropical areas of South America such as Brazil suffer from the highest burden of CT worldwide due to the presence of highly virulent toxoplasma genotypes, high numbers of infected felines and a climate facilitating oocyt survival [2, 46]. Brazil also plays an important role in the global research activity on other infectious and particularly parasitic diseases [47]: In a study on yellow fever, Brazil ranked in position two with 203 publications after the US (751 publications) and was followed by France (149 publications) and the UK (113 publications) [35]. A similar picture was also present for leishmaniasis. Here, two studies assessed the research output for the periods between 1957 and 2006 and 1945 and 2010 [48, 49]. Most publications came from high-income countries such as the US, UK or Germany, but Brazil was also found among the top leading countries [48]. For our study, we want to stress that no other South American nation besides Brazil (e.g. Columbia or Argentina) played an outstanding role regarding global CT research output. Brazil did not only produce more but also higher cited publications - and thus research of more advanced scientific quality and recognition - than countries like Columbia, Argentina and Chile (as shown in Fig. 2a, b and c). Also, the impact of Eastern Mediterranean, and African regions was marginal, although all have high incidences of CT (up to 3.4 CT cases per 1000 live births according to the WHO) [50].

The relation of CT research activities to economic data indicated that Switzerland, France, the UK and Brazil invested the highest overall resources and gained the highest research output when toxoplasmosis publication activity was related to absolute numbers of GDP. However, when the toxoplasmosis research activities were evaluated taking manpower into account and related to the per capita economic power (GDP/capita), the picture changed and the US hold the first position followed by Brazil; Switzerland ranked last. This analysis of research output in relation to economic benchmarks further illustrated the accentuated position of Brazil among the key players of CT related research. We found that Brazil - although an upper-middle-income country among high-income countries - ranked second after the US when CT-specific publications were related to the relative economic power (measured in GDP per capita in 1000 US-$). Here, 85.95 CT-related publications were produced per GDP per capita in 1000 US-$ indicating that this country invests a relatively high proportion of its resources very effectively in toxoplasmosis research. Overall, it can be stated that countries with high GDP should have the capability to invest larger sums in research and development, and thus can create more attractive conditions for science. However, the GDP should always also be considered in relation to the population, which can change the picture. In this respect, China is an impressive example in our study.

When focussing on subject categories in toxoplasmosis research, we identified “Parasitology”, “Immunology”, “Microbiology” and “Veterinary Science” as the leading areas regarding publication and citation numbers. This finding was not surprising. In this context, we want to highlight that a rather small quantity of articles published in the category “Immunology” received an extremely high number of citations pointing towards their significant scientific quality. Regarding the chronological evolution of categories, we documented that the relative percentage of items published in “Tropical Medicine” and “Microbiology” decreased from 1963 onwards. We attribute this development to the fact that research on T. gondii advanced over time shifting its focus from identifying and locating the parasite to specific investigations regarding its life cycle, transmission patterns and clinical implications. This shift in scientific interest correlated with an increased percentage of published items found in other categories, e.g. in “Immunology” since 1968. The rising interest in this particular area might be explained by the onset of serologic testing – still the corner stone for diagnosis of CT today [20, 51–53] - in the 1960s based on the description of antibody structures by Cohen and Porter [54]. During the 1970s, we documented a visible growth of publication activity in “Cell Biology”. This also corresponds with the historical milestones at this time: Researchers decoded the invasion processes, life cycle of the parasite and transmission patterns with the help of cell biological methods. Also, the molecular biological method of polymerase chain reaction (PCR) was developed in 1987 [55]. Since PCR plays a crucial role in fetal diagnosis of CT [1, 56, 57] and allows to characterize different T. gondii genotpypes, which became increasingly relevant in the 90s [12, 20], this technique might have contributed significantly to growing volume of publications in the area of “Biochemistry and Molecular Biology”. In our analysis, it became evident that no publications were attributed to the category of “Public Health”. Since CT has an tremendous global impact on the health of mothers and their offspring, our finding illustrates the apparent need to increase the worldwide implementation of Public Health measures and their assessment [15]. Furthermore, the relatively small proportion of toxoplasmosis research in the field of pharmacology indicates that this area should receive more attention in future.

France and Brazil were identified as nations with more female than male authors participating in CT-associated research. A similar gender distribution was found for Rotavirus-related research [58]. The authors explained their results with the Brazilian culture of encouraging female scientists to particularly participate in economy, science and technology as shown by three large benchmarking studies [58, 59]. However, we could identify only 48.8 % of author names regarding their gender. Common difficulties were encountered when names were documented as initials, were gender neutral and not listed in accessable name databases. Hence, this part of our study has to be viewed critically.

On international level, we found a growing number of collaborations since 1973, which was congruent with an increasing number of authors per paper. This development might be related to the overall trend to conduct research as collaborative efforts but also be based on emerging, well funded research networks such as the international “Systematic Review On Congenital Toxoplasmosis (SYROCOT) study group” or the “European Research Network on Toxoplasmosis” [14]. We identified the US as the nation involved in the highest number of international collaborations – most of these were established between the US and the UK, France and Germany. This picture corresponds to other studies [60] and can be explained with the excellent scientific infrastructure and the majority of institutions working on CT-related research based in the US. Also, Brazil issued 262 of its 973 published paper in conjuction with US American scientists. This finding is remarkable and can be explained by the dedicated support of the Brazilian government and the unique opportunities researchers gain in regards to the specific T. gondii populations endemic in South America [46].

The infection of pregnant women and unborn children still challenge healthcare providers from a global perspective. Hence, we want to point out the necessity to include low-resource countries with high CT incidence rates (e.g. located in Central/South America or the Eastern Mediterranean [50]) in strong scientific collaborations. Here, epidemiological data, ideas and gained knowledge can be exchanged to the benefit of all participants. In this context, we want to underline the ethical responsibility to involve African countries in collaborative efforts. Currently, these nations play no visible role within the global CT research community but should participate since they have not only high prevalences of CT but also a large demographic of toxoplama-susceptible women with HIV and no adequate therapy (e.g. Ethopia) [61]. Furthermore, collaborations should focus on including countries with access to vast epidemiological data from prenatal and neonatal CT screening or surveillance programs such as France, Austria, Italy, Denmark, Poland [7]. Since global travel increases, atypical parasites from South America will also become more relevant for Europe. Here, close-knit networks and shared public health efforts allowing the exchange of epidemiological data, ideas and resources are essential for effective prevention.

Regarding the strengths of our study, we employed the WoS for raw data acquisition due to its unique feature to generate detailed citation for single index entries. These enabled us to calculate semi-qualitative variables such as country-specific citation rates or h-Indices, which added valuable aspects to our assessment. Also, CT is a newly researched field. Hence, using the WoS provided us with the particular opportunity to investigate the complete chronological development of the related research output. Nevertheless, one bias of our study is linked to the WoS’s focus on English publications. Hence, non-English items – particularly when published before 1940 – might be under-represented in our analysis. Also, we hypothesize that articles written in Spanish and Portuguese describing early research conducted in countries with high T. gondii prevalence such as Colombia, Argentina or Brazil might not have been identified by our search; the Scielo bibliographic database would be an appropriate source to investigate these. On the other hand, this language bias might be limited since Thompson Reuters stated that 90 % of cited and 75 % of published items related to a specific topic are indexed by the WoS. Also, non-English speaking countries usually publish their high quality research in English journals [62]. Another bias might be related to the citation parameters serving as proxies for scientific quality. Although this relationship between citation numbers and scientific quality is well established, it might be skewed due to the common habit of self-citing and the Matthew effect. Here, publications issued by well known scientists are cited more than junior scientists leading to an exponential growth of citation counts [63].

## Conclusions

In summary, this study is the first in depth analysis of the worldwide toxoplasmosis research architecture. Using density-equalizing mapping techniques, a global landscape was sketched representing quantitative and qualitative toxoplasmosis research aspects. The US has a leading role in the field followed by other high-income countries. Brazil occupied an emphasized position in the scientific community, which reflects the country’s dedication to CT- related research to fight a disease that puts a major burden on the health of its inhabitants. Also, our study highlights the apparent need to conduct research in the field of Public Health: Interventions should be tailored to the continent since the disease presents differently in South America than Europe. Also, we have to update our educational efforts to inform about potential exposure to highly viruent T. gondii genotypes due to traveling or consumption of imported foods.
